# 10-Year Mortality Pattern Among Cancer Patients in Lagos State University Teaching Hospital, Ikeja, Lagos

**DOI:** 10.3389/fonc.2020.573036

**Published:** 2020-11-30

**Authors:** Omolara Aminat Fatiregun, Omowunmi Bakare, Sunday Ayeni, Adebowale Oyerinde, Anthonia C. Sowunmi, Abiodun Popoola, Omolola Salako, Adewumi Alabi, Adedayo Joseph

**Affiliations:** ^1^Department of Radiology & Oncology, Lagos State University, Ojo, Nigeria; ^2^Department of Radiology & Oncology, Lagos State University College of Medicine, Ikeja, Lagos; ^3^Department of Community Health and Primary Health Care, College of Medicine, Lagos State University, Lagos, Nigeria; ^4^Department of Medical Records, Lagos State University Teaching Hospital, Ikeja, Nigeria; ^5^Research Department, Cancer Explore Foundation, Lagos, Nigeria; ^6^Department of Radiation Biology, Radiotherapy, Radio-diagnosis and Radiography, College of Medicine, University of Lagos, Lagos, Nigeria; ^7^LUTH/NSIA Radiotherapy Centre, Lagos University Teaching Hospital, Lagos, Nigeria

**Keywords:** mortality, patterns, cancer patients, cancer related, Lagos state, cancer deaths

## Abstract

**Background:**

Globally, cancer is a major leading health problem with an estimated 10 million incidences and 6 million cancer deaths annually. In Nigeria, an estimated 72,000 cancer deaths occur annually, and 102,000 new cases are diagnosed from its population of 200 million people. These are, however, estimates, it is necessary to document the yearly trends and patterns of cancer mortality with regards to the different regions in the country.

**Methodology:**

we conducted this study at the Lagos State University Teaching hospital (LASUTH), Ikeja, Lagos to document mortality patterns from 2009 to 2018. Data extracted included those from the patient’s case notes, admission and death registers, and death certificates. we also had records from the hospital records department and medical wards. We then documented cancer mortality over the study period.

**Results:**

A total number of 6,592 deaths were recorded over ten years, and 1,133 cases were cancer-related deaths. This number puts the percentage of cancer-related deaths at 17.2%. Male patients accounted for 54.0%, and female patients are 46.0%. Breast cancer accounted for the highest mortality, followed by prostate cancer. The highest number of deaths were recorded in 2010 at 821, followed by 2011 at 799, 2015 at 780, and the least in 2017 at 513. There is also a significant general increase in odds of mortality with an increase in decades of life.

**Conclusion:**

This study shows that about one in five deaths, over the last ten years, from this tertiary institution, is related to a cancer diagnosis. Even though a yearly decline in the number of cancer deaths was noticed, probably due to increased awareness and governmental intervention, the percentage still remains high.

## Introduction

Cancer is the world-leading cause of death, cancer mortality rates are more than deaths caused by HIV/AIDS, tuberculosis, and malaria put together. It is the second leading cause of death in developed regions and is among the three most causes of death for adults in developing regions (1–5). It estimates for 7.6 million deaths (about 13% of all deaths) in 2008 and is projected to continue increasing, with an account of 13.1 million deaths in 2030 (4). In 2002, there were 6.7 million world cancer deaths, with less than 5% of these in sub-Saharan Africa. Still, it has been accounted that, by 2020, cancer could lead to the death of 10.3 million people worldwide, with a 50 to 75% rise in cancer death in sub-Saharan Africa (4). Cancer is one of the most common non-communicable diseases and has become an essential contributor to the global burden of diseases. The burden of cancer is rising, and it is one of the most causes of death worldwide (6).

The cancer mortality pattern is quite different in Africa when compared to other parts of the developed world. In 2012, there were an estimated 626,400 new cases of cancer and 447,700 deaths from cancer in Sub-Saharan Africa. cancer incidence in Sub-Saharan Africa is projected to rise by 85% in the next fifteen years. Cancer in Africa is characterized by late diagnosis and presentation, low access to treatment, and poor treatment outcomes. Inadequate access to cancer treatment results in 80–90% of cases that are in an advanced stage to result in death.

Cancer is responsible for 72,000 deaths in Nigeria annually, with an accounted 102,000 (7) new cases of cancer annually. In Nigeria, with a population of nearly 200 million people, complex diseases such as cancer are currently emerging as critical health care priority for the future. The data available on cancer mortality is inadequate in Nigeria, especially with regards to yearly trends and patterns of cancer mortality with regards to the different regions and states in the country. This study was conducted to provide data on the patterns of cancer mortality in Lagos state university teaching hospital, LASUTH over ten years using the data obtained from the hospital death certificates and death registers.

## Methodology

This study is a retrospective study in which the cancer deaths (outcomes) have already occurred. Data were extracted from patient’s case notes, admission and death registers, and death certificates, retrieved from the hospital records department and medical wards. These were reviewed to document the cause of death. These data include patient demographic data age, sex, clinical information, and histopathological type of cancer, year of death, and data were analyzed according to sex and age distribution for all cases. Ethical clearance was obtained from the hospital’s ethics committee before the commencement of study.

Analysis of the data was done using the Statistical Package for Social Sciences (SPSS version 22.0). Simple descriptive statistics were used. The data was analyzed statistically using simple figures, ratio, percentages, table, and graphs. Mean, and Standard deviation was applied for continuous variables. Inferential statistics included logistic regression to explain the relationship between variables, and P-value 0.05 was taken to be statistically significant.

## Results

**Figure 1:**
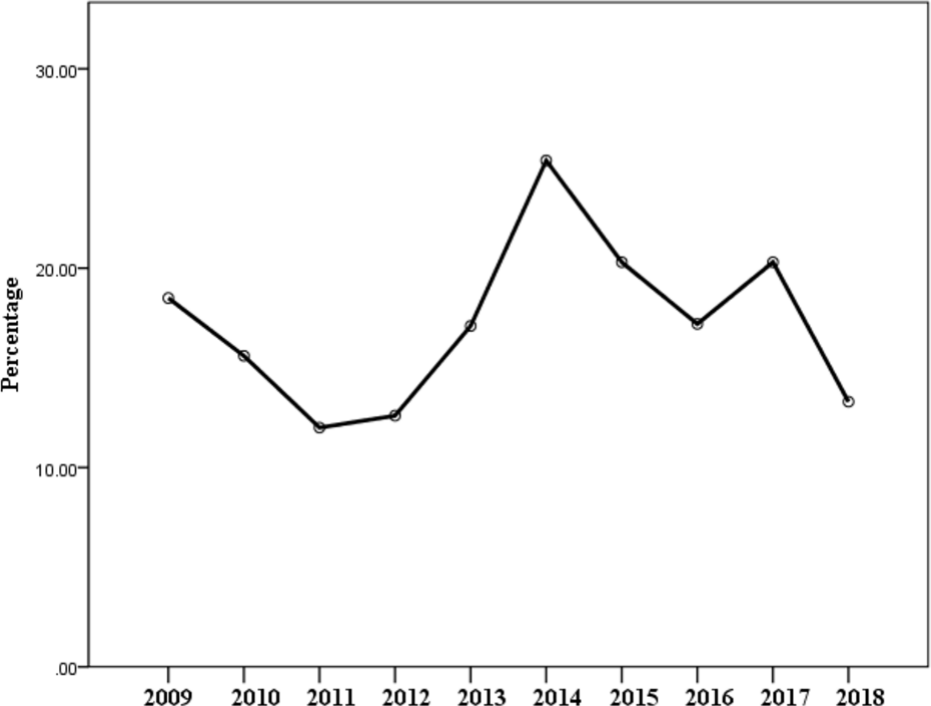
A graph illustrating the yearly cancer mortality.

This study aimed at providing data on the pattern of cancer mortality in Lagos State University Teaching Hospital, LASUTH. A total of 6592 deaths were recorded over ten years, with 1,133 being cancer-related deaths. This number puts the percentage of cancer-related deaths at 17.2% (**Figure 1**). It is observed that out of all deaths that occurred during the study, male patients accounted for 54.0%, and female patients are 46.0%. Mean age of cancer mortality for both ages was 51.3 ± 10.9 (**Table 1**). Based on age group as a variable, 50–59 and 60–69 as well as <10 at 15.5, 17.7, and 14.1% respectively have a high mortality pattern (**Table 1**). Most male and female deaths occurred between ages 60-69 and 50-59 respectively (**Table 2**). The highest number of deaths were recorded in 2010 at 821, followed by 2011 at 799, 2015 at 780, and the least in 2017 at 513 (**Table 3**). Mean age of cancer mortality for both ages was 51.3±10.9 (**Table 4**).

**Table 1:** Showing gender and age distribution of mortality pattern over ten years.

Variable	Frequency (n = 6,592)	Percentage
**Gender**		
Male Female	3,559 3,033	54.0 46.0
**Age group (Years)**		
<10 10–19 20–29 30–39 40–49 50–59 60–69 70–79 80–89 ≥90	931 247 340 716 888 1,019 1,167 898 336 50	14.1 3.7 5.2 10.9 13.5 15.5 17.7 13.6 5.1 0.8

**Table 2:** Showing age and gender distribution with regards to cancer mortality.

Variable	Male (n = 531)	Female (n = 602)	p-value
**Age group (Years)**			
<10 10–19 20–29 30–39 40–49 50–59 60–69 70–79 80–89 ≥90	35(6.6) 17(3.2) 20(3.8) 40(7.5) 74(13.9) 90(16.9) 125(23.5) 99(18.6) 20(5.6) 1(0.2)	17(2.8) 17(2.8) 21(3.5) 68(11.3) 118(19.6) 145(24.1) 127(21.1) 69(11.5) 17(2.8) 3(0.5)	0.108

**Table 3:** Showing yearly mortality pattern.

Variable	Frequency (n = 6,592)	Percentage of Cancer cases
**Year**		
2009 2010 2011 2012 2013 2014 2015 2016 2017 2018	541 821 799 571 604 689 780 711 513 563	8.2 12.5 12.1 8.7 9.2 10.5 11.8 10.8 7.8 8.5

**Table 4:** Showing the sex and age distribution for cancer and non-cancer mortality.

Gender	Cancer (n = 1,133)	Non-cancer (5,459)	p-value
			<0.001
Male Female	531(14.9) 602(19.8)	3,028(85.1) 2,431(80.2)	
**Age group (Years)**			<0.001
<10 10–19 20–29 30–39 40–49 50–59 60–69 70–79 80–89 ≥90	52(5.6) 34(13.8) 41(12.1) 108(15.1) 192(21.6) 235(23.1) 252(21.6) 168(18.7) 47(14.0) 4(8.0)	879(94.4) 213(86.2) 299(87.9) 608(84.9) 696(78.4) 784(76.9) 915(78.9) 730(81.3) 289(86.0) 46(92.0)	
Both sexes MeanAge ±SD Mean Age for Males Mean Age for Females	51.38 ± 10.9 51.73 ± 10.8 50.97 ± 11.1		

**Figure 2:**
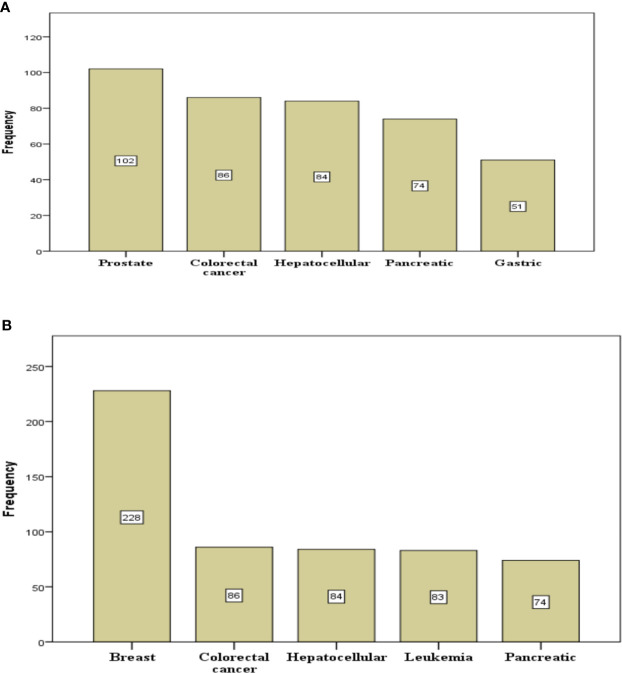
Five commonest cancer-related mortality in male **(A)** and female **(B)** over ten years.

Of the total number of deaths recorded, male cancer patient’s death was 14.9% while male non-cancer death was 85.1% and female cancer patient’s death was 19.8% while female non-cancer patient’s death was 80.2%. Cancer deaths were commoner in female compared to male (p < 0.001), as shown in **Table 4**. Among cancer-related deaths, male patients accounted for 46.9%, and female patients accounted for 53.1%. Cancer mortality was observed in different age groups as follows; 40–49 (21.6%), 50–59 (23.1%), 60–69 (21.6%), and 70–79 (18.7%) and the least was >90 (8.0%). **Table 4** compares yearly cancer and non-cancer mortality pattern; in 2011, cancer-related deaths recorded were 96 (12.0%) and non-cancer-related deaths were 703 (88.0%), also, in 2017, cancer-related deaths recorded were 104 (20.3%) and non-cancer-related deaths were 409 (79.7%). The highest number of cancer deaths were recorded in 2014, 175 deaths, followed by 2015, 158 deaths, 2010, 128 deaths, 2015, 122 deaths and the least in 2012, 72 deaths. The yearly number of cancer-related deaths ranged between 12.0% (2011) to 25.4% (2014) (illustrated in **Figure 1** and **Table 5**), while yearly non-cancer-related deaths ranged between 74.6% (2014) to 88.0% (2011). The highest peak, as illustrated in **Figure 1**, depicts the highest number of cancer deaths recorded at 25.4%, 2014 and the lowest peak, the least number of deaths at 12.0% in 2011. Breast cancer was responsible for most of the deaths and accounted for 228 (20.1%) (**Table 6**), followed by prostate cancer which accounted for 102 deaths (9.0%). Colorectal cancer, hepatocellular, leukemia, and pancreatic cancer were responsible for 86 (7.6%), 84 (7.4%), and 86 (7.3%) respectively. Five commonest causes of cancer mortality are as depicted in **Figure 2**. In females, breast cancer was the commonest, followed by colorectal, hepatocellular, leukemia and pancreatic. For males, the commonest was Prostate cancer, followed by colorectal, hepatocellular, pancreatic and then gastric (**Figure 2**). Females had increase odd (1.447 95% CI = 1.270–1.648, p < 0.001) of dying when compared with males in this study. There is also a significant increase in odds of mortality with an increase in decades of life (**Table 7**). However, reduce odds was noted in terminal ages likely due to other factors associated with mortality in those age group (p < 0.001).

**Table 5:** Showing yearly cancer and non-cancer mortality rate.

Year	Cancer (n = 1,133)	Non-cancer (5,459)	p-value
			<0.001
2009 2010 2011 2012 2013 2014 2015 2016 2017 2018	100(18.5) 128(15.6) 96(12.0) 72(12.6) 103(17.1) 175(25.4) 158(20.3) 122(17.2) 104(20.3) 75(13.3)	441(81.5) 693(84.4) 703(88.0) 499(87.4) 501(82.9) 514(74.6) 622(79.7) 589(82.8) 409(79.7) 488(86.7)	

**Table 6:** Showing Organ-specific mortality in Males and Females over ten years.

	Male (n = 531)	Female (n = 602)	Total
Breast	5(0.9)	223(37.0)	228(20.1)
Prostate	102(19.1)	0(0.0)	102(9.0)
Colorectal cancer	47(8.9)	39(6.5)	86(7.6)
Hepatocellular	54(10.2)	30(5.0)	84(7.4)
Leukemia	44(8.3)	39(6.5)	86(7.3)
Pancreatic	40(19.1)	34(5.6)	74(6.5)
Gastric	25(10.2)	26(4.3)	51(4.5)
Lymphoma	29(5.5)	20(3.3)	49(4.3)
Renal	22(4.2)	9(1.5)	31(2.7)
Ovarian	0(0.0)	30(5.0)	29(2.6)
Intraabdominal	14(2.6)	14(2.3)	28(2.5)
Bile duct	13(2.5)	10(1.7)	23(2.0)
Myeloma	13(2.5)	7(1.2)	20(1.8)
Thyroid cancer	1(0.2)	4(0.7)	5(0.4)
Stomach	5(0.9)	3(0.5)	8(0.7)
Esophageal	8(1.5)	4(0.7)	12(1.1)
Skin	6(1.1)	3(0.5)	9(0.8)
Anorectal	5(0.9)	1(0.2)	6(0.5)
Bladder	10(1.9)	5(0.8)	15(1.3)
Rhabdomyosarcoma	11(2.1)	5(0.8)	16(1.4)
Sacrococcygeal teratoma	0(0.0)	2(0.3)	2(0.2)
Rectum	8(1.5)	8(1.3)	16(1.4)
Gall bladder	3(0.6)	9(1.5)	12(1.1)
Wilms tumor	1(0.2)	0(0.0)	1(0.1)
Endometrial	0(0.0)	11(1.8)	11(1.0)
Thymoma	2(0.4)	1(0.2)	3(0.3)
Lung	12(2.3)	7(1.2)	19(1.7)
Glioblastoma	2(0.4)	4(0.7)	6(0.5)
Nasopharyngeal	2(0.4)	1(0.2)	3(0.3)
Neuroblastoma	1(0.2)	1(0.2)	2(0.2)
Periampullary	1(0.2)	2(0.3)	3(0.3)
Neck	2(0.4)	0(0.0)	2(0.2)
Cervical cancer	1(0.2)	12(2.0)	13(1.1)
Laryngeal cancer	4(0.8)	1(0.2)	5(0.4)
Bronchogenic carcinoma	11(2.1)	5(0.8)	16(1.4)
Brain	3(0.6)	6(1.0)	10(0.9)
Others	24(4.5)	25(4.2)	50(4.4)

**Table 7:** Logisticregression showing socio-demographic predictors of mortality.

	Odd ratio	95% CI	p-value
**Gender**			
Male Female	1 1.447	1.270–1.648	<0.001
**Age group (Years)**			
<10	1		
10–19 20–29 30–39 40–49 50–59 60–69 70–79 80–89 ≥90	2.673 2.265 2.929 4.673 5.090 4.684 3.915 2.727 1.394	1.694–4.226 1.473–3.484 2.070–4.144 3.384–6.452 3.711–6.981 3.425–6.405 2.824–5.427 1.797–4.137 0.482–4.030	`<0.001 <0.001 <0.001 <0.001 <0.001 <0.001 <0.001 <0.001 <0.001

## Discussion

There is a rising trend in the incidence of cancer in Nigeria (8). Most patients present in a late-stage which leads to poor treatment outcomes, poor prognosis and increased cancer mortality. This study showed the pattern of cancer mortality in Lagos State University Teaching Hospital, LASUTH over ten years. Cancer deaths accounted for 17.2% of all the deaths in the hospital over the study period. The pattern is high when compared with other studies done in Africa, a study done in Tanzania showed that only 5.1% of deaths over ten years was cancer-related (9–11). The mortality pattern due to cancer in Africa is rising. In sub-Saharan Africa, cancer deaths have increased by 45 percent since 2000, with yearly mortality of more than half a million people (5, 12). Other attributable might include differences in climate, diet, genetic factors, development rate, and some other unknown factors (10, 11).

In 2011 and 2012, it was observed that the number of cancer deaths was low, but the number increased steadily in 2013 and 2014. From 2015 and into the following years, the number of deaths significantly decreased. This initially increases in cancer deaths in 2013 might be due to increased hospital presentations and reporting of cancer patients and cases, because of the commencement of Lagos state Ministry of Health cancer programs. More cancer patients presented to the LASUTH, which is a referral center for most state programs conducted at the Primary and secondary care levels and the only state-owned tertiary hospital offering tertiary cancer treatment in Lagos state. On the other hand, the reduction of deaths from 2015 onwards shows the impact of these screening programs conducted by the state government and some non-governmental organizations in Lagos State. As more patients presented with earlier disease, and fewer deaths were recorded, these numbers are however, still very high (13).

The mean age was 51.3 years, and the highest incidence of cancer deaths was seen in the age group 60–69. However, the age range of 50–59 and 40–49 also had very high incidence, and the pattern is similar to that reported by Akinde et al. (11), most mortality cases are seen in their study were between 51 and 60 years. The range of patients seen in this series falls within the stated life expectancy of Nigerians, which is 55 years and 56 years for males and females respectively, according to the WHO (14). Cancer mortality was observed to be higher in females at 53.1% compared to males, 46.9% giving a female to male ratio of 1.1:1. This is almost equal to the 1.2:1 female to male ratio in Kano Cancer Registry (KCR) (15). Reports from developed countries showed virtually identical or slightly increased M: F ratio as cancers take their toll in both sexes almost equally (16). The difference is noteworthy and can be proposed to be caused by the increased occurrence of breast cancer. In order of increasing frequency, organ-specific cancer mortality observed were, breast cancer, followed by prostate cancer, colorectal cancer, hepatocellular cancer and leukemia. This pattern is similar to the one observed in the University of Port Harcourt Teaching Hospital (UPTH) which was breast cancer, ranked first, followed by prostate cancer and hematolymphoid cancer while colorectal cancers ranked 4^th^ (17). Among the least common were neck cancer, neuroblastoma, thymoma, nasopharyngeal and thyroid cancer.

Breast cancer is the commonest cause of cancer deaths recorded in this study, accounting for 228 deaths (20.1%). Global estimates for 2012 has revealed 1.67 million breast cancer cases worldwide ranking it as the second most common malignancy (18). Breast cancer is the most commonly diagnosed cancer in Africa and Sub−Saharan Africa and is also the leading cause of death from cancer (63,100 deaths in 2012) (18). Breast cancer carries a massive burden on the nation. Breast cancer mortality poses a severe public health threat in Nigeria and indeed, in most countries of the world (19). In our opinion, one key factor that, plays a crucial role in breast cancer mortality in our study is a late stage of presentation (20). often a consequence of poverty, ignorance, and inaccessible health care facilities.

Prostate cancer is the commonest cancer in males in Nigeria and Sub−Saharan Africa (2), it accounted for 9.0% deaths in this study, this is lower than the 13% in a South African study (21) and almost equal to the rate of 9.2% of mortality cases recorded in University of Port Harcourt Teaching Hospital (UPTH), Nigeria (17). According to GLOBOCAN 2012 estimates, prostate cancer ranked as the most common cancer in males worldwide with increasing survival rates due to screening programs available in most developed countries. The 5-year survival rates in the USA for men diagnosed with prostate cancer is around 98% (22) while the data from Eurocare project (EUROCARE-5) from 2003 to 2007 showed that 5-year survival rate was 83% (23). More cases of prostate cancer will be diagnosed at an early stage if routine screening is available in Nigeria to screen men.

Colorectal cancer accounted for 7.6% deaths in this study; this result is like the 7.2% recorded in the Kano Cancer Registry (KCR) (15). Colorectal cancer is the third most common cancer in men and the second in women worldwide. The highest incidences are seen in the developed world while the lowest is noted in Africa, where it ranked as the fifth most common malignancy (2).

Carcinoma of the cervix accounted for 1.1% of cases in this study, this is low compared to global mortality and might indicate under-reporting of mortality cases from the disease, which is a significant challenge of documenting cancer patterns in Nigeria. Another attributable factor is that of a regional difference in the mortality of this disease in this state. Even though there is a policy on cancer control in Nigeria, the National Cancer Control Plan, (7) this policy has to be implemented effectively in other to reduce the burden of cancer on the nation. Currently, only 0.18% of the health care budget is allocated to cancer-related programs in Nigeria (24). The National budgetary allocation for health is also meager (25). The budgetary allocation for healthcare in 2020 was 4.3% of the total budget, as compared to other parts of the world, for instance, the US in 2020 dedicated about 21% (mandatory and discretionary spending’s on health) (26) and the UK allocated about 19% of their total budget to healthcare. Also, there is an urgent need to increase funding and widen the coverage of these programs directed towards cancer prevention, screening services for prompt diagnosis, and optimal treatment services.

### Limitations

A major limitation of this study was the challenge of poor record keeping in the most centers in Nigeria, data collection is still paper based which is prone to loss of records. Also, the cancer registry is poorly funded and understaffed hence the need to include records from other sources like the death certificates and registers for this review.

## Conclusion

Cancer mortality in Nigeria is still high. This study shows that about one in five deaths, over the last ten years, from this tertiary institution, is related to a cancer diagnosis. Breast cancer, which is more predominant in females, accounted for the highest mortality followed by prostate cancer which accounted for increased mortality in males. Even though a yearly decline in the number of cases was documented in this study, the percentage remains high. The decrease in the mortality pattern of cancer patients noticed in the last five years, shows the impact of the increased awareness, government intervention as well as regular screening for early detection and the practice of self-breast examination. However, there is still much that needs to be done, both locally and nationally, to reduce further the burden documented in this study.

## Data Availability Statement

The raw data supporting the conclusions of this article will be made available by the authors, without undue reservation.

## Ethics Statement

Ethical clearance was obtained from the Lagos State University Teaching Hospital ethics review committee, REF NO: LREC/06/10/1142.

## Author Contributions

OF, AS, and AP were involved in conceptualization and development of research idea. AO, SA, and OB were involved in data extraction and statistical analysis. AJ, AA, and OS were involved in final manuscript editing. All authors contributed to the article and approved the submitted version.

## Conflict of Interest

The authors declare that the research was conducted in the absence of any commercial or financial relationships that could be construed as a potential conflict of interest.
